# Analysis of the Capacity of Google Trends to Measure Interest in Conservation Topics and the Role of Online News

**DOI:** 10.1371/journal.pone.0152802

**Published:** 2016-03-30

**Authors:** Le T. P. Nghiem, Sarah K. Papworth, Felix K. S. Lim, Luis R. Carrasco

**Affiliations:** 1 Department of Biological Sciences, National University of Singapore, 14 Science Drive 4, Singapore 117543, Republic of Singapore; 2 Royal Holloway, University of London, Egham Hill, Egham, Surrey TW20 0EX, United Kingdom; Tianjin University, CHINA

## Abstract

With the continuous growth of internet usage, Google Trends has emerged as a source of information to investigate how social trends evolve over time. Knowing how the level of interest in conservation topics—approximated using Google search volume—varies over time can help support targeted conservation science communication. However, the evolution of search volume over time and the mechanisms that drive peaks in searches are poorly understood. We conducted time series analyses on Google search data from 2004 to 2013 to investigate: (i) whether interests in selected conservation topics have declined and (ii) the effect of news reporting and academic publishing on search volume. Although trends were sensitive to the term used as benchmark, we did not find that public interest towards conservation topics such as *climate change*, *ecosystem services*, *deforestation*, *orangutan*, *invasive species* and *habitat loss* was declining. We found, however, a robust downward trend for *endangered species* and an upward trend for *ecosystem services*. The quantity of news articles was related to patterns in Google search volume, whereas the number of research articles was not a good predictor but lagged behind Google search volume, indicating the role of news in the transfer of conservation science to the public.

## Introduction

Support from the public is often a prerequisite for conservation success [1, 2], thus understanding the dynamics of public interest is crucial for effective conservation initiatives. Traditionally, public surveys have been used to gauge levels of awareness or support from the public towards environmental causes (e.g. [3], [4]). More recently, internet usage has emerged as an extensive source of data to investigate public trends. Some examples are the use of social media as an indicator of public interest in environmental topics [5], public health outcomes [6] and the use of Google search volume to predict economic trends [7] and disease outbreaks [8]. Online data from Google have become a valuable source for analysis as Google is the most popular search engine globally (Google constituted over 65% of the global online search share in 2012 [9]). Conservation scientists have started to explore the potential of Google data, such as linking search volumes with unsolicited donations to a conservation charity [10] or measuring public interest in bird and butterfly species [11]. The use of Google Trends can provide time-series information on fluctuations in public interest in numerous topics, especially across fine temporal (weekly) and spatial scales across the globe. Analysing how public interest in conservation issues changes with time and the factors that drive these changes could provide understanding of how information is communicated to the public, and may help inform decisions regarding conservation science communication.

Recent studies using Google to explore public interest in conservation issues over time have yielded inconclusive findings: while a declining interest in environmental issues was reported (e.g. [12]), these results have been contested [13] on technical grounds. The main reason behind technical criticisms is that absolute Google search volume is not reported. Instead, search volume is presented as a percentage relative to the peak search volume obtained during the specified time period and scaled by the total search volume for each specific term [7]. Therefore, as new search terms are constantly added to Google over time, a shrinking relative share of search volume for individual terms will be reported. In theory, this natural shifting baseline could be captured by “neutral terms” that are not expected to have changes in interest associated to new uses of the internet [13]. In addition, applying time series analyses could also help to gain insights into the effects and changes that may not happen immediately but require a time lag to be realized [14, 15].

Additionally, previous studies on public interest in conservation topics have not accounted for possible drivers of search interest such as news reporting and academic publishing. News media can influence public opinion on environmental and socio-political issues, such as the threat of climate change [16], foreign policies [17] and public stigmas of obesity [18]. Media-content analysis is a recent research focus for conservation scientists, demonstrated by research on media coverage of the Florida panther [19], sharks [20], and human-leopard conflict [21].

Academic publishing is generally considered to play an educational role to the public. The “deficit model” describes communication as a top-down process where scientists communicate information to the knowledge-deficit public for consumption [22]. Even though this top-down approach has been criticized [23], a lag time is often assumed between scientific findings and assimilation of information by other stakeholders [24]. Therefore, understanding the link between academic publishing and Google searches on conservation topics can provide important information to target communication of conservation science to the public.

To advance understanding of the potential for Google Trends to gauge online interest in conservation topics and identify drivers of changes in search volume, this study uses trend analysis and time series analysis to: (i) study Google search trends in selected conservation topics to assess the effect of controlling for the shifting baseline in Google Trends using different benchmark keywords; and (ii) explore the temporal relationships between search volumes reported by Google Trends, news coverage, and research articles in conservation biology.

## Methods

### Google Trends dependent variables

To examine the effect of number of research articles and news coverage on public interest in conservation topics by the online public, we selected specific conservation-themed keywords. The criteria for selecting keywords were: (i) words or phrases representing major areas of research in conservation biology; (ii) words or short phrases which are specific and not prone to confusion with other popular, non-conservation search keywords (e.g. *tiger* or *PES* (payment for ecosystem services) may be used to refer to a famous golfer or a popular video game respectively, so were excluded); (iii) overlap with previous studies looking at online interest in environmental topics ([12], [13]); and (iv) words or phrases with sufficient search volume in Google Trends to facilitate the analysis (e.g. *wildlife consumption* was discarded for this reason). The final seven keywords selected were: *climate change*, *ecosystem service* (or *ecosystem services)*, *deforestation*, *orangutan* (or *orang-utan*), *invasive species*, *endangered species*, and *habitat loss*. Though these keywords did not purport to be a comprehensive representation of conservation topics, each keyword represents a real-world, topical conservation issue that can be illustrative for our approach.

We used the search volume in Google Trends as a proxy for changes in public interest across a 10-year period, starting from January 2004 (when Google Trends data were first available) until December 2013. Search volume in Google Trends is the traffic for a specific keyword relative to all queries submitted in Google, normalized to range from 0 to 100, with 100 corresponding to the peak of relative search volume obtained for each keyword during the period of interest [7].

To correct for shifting baselines in overall search volume, we transformed relative monthly search volume for each keyword by dividing it by a benchmark term. We chose four terms spanning a range of non-decreasing to increasing interest to assess the sensitivity of this correction to the choice of benchmark term. The benchmark terms selected were: *software*, *computer*, *life* and *love*. The choice of these terms is based on suggestions from previous studies. Actual public interest in *software* and *computer* presumably remains consistent and these two terms can be used as neutral terms [13]. *Love* and *life*, on the other hand, are popular terms with presumably increasing popularity in the social web and news archives [25] and used as benchmark keywords in related research on public interest in the environment across different languages [26]. Statistical analyses were conducted on the benchmark-corrected data in the R environment [27].

### Trend analysis

We used seasonal Mann-Kendall tests in the package Kendall [28] to detect trends in Google search volume over 10 years. The Mann-Kendall test is a non-parametric test to detect trends in time series analysis when the series are not normally distributed [29], as was the case for our data. An alternative non-parametric test for trend detection is Spearman’s rho test, which yields similar results [30]. The Mann-Kendall test tests the data against a null hypothesis of no trend and calculates Kendall’s tau statistic *τ* based on *S*, the subtraction of the discordant pairs (*x*_*j*_<*x*_*k*_ for *j*>*k*, where *x* denotes the variable of study and *j* and *k* denote current and future points in time respectively) from the number of concordant (x_j_>x_k_ for j>k) pairs across all possible pairs in all the *n* observations in the time series. *τ* is calculated as:

$$\tau =\frac{S}{\frac{1}{2}n(n-1)}$$

A positive or negative value of S (and thus *τ*) indicates an upward or downward trend respectively [29]. The seasonal Mann-Kendall test accounts for monthly variation in the data by calculating separate *S* for each month of the year [31].

### Time series analysis of relative search volume as a function of news and research articles

#### Data collection

For news articles, we queried LexisNexis Academic, which hosts a global online electronic library of public records, for all English news using each of the 7 keywords as the search term. For scholarly articles, we queried Web of Science for scholarly articles in the fields of environmental sciences, ecology, environmental studies, and biodiversity conservation. We used each of the 7 keywords as search topics. All raw data collected were in monthly intervals (e.g. monthly search volume in Google, monthly news articles recorded in LexisNexis, and monthly scholarly articles in Web of Science) from 2004–2013, resulting in a total of 120 data points for each keyword.

#### Identification of lags in the explanatory variables

Since previous studies have shown that the relationship between the variables considered may not be contemporaneous [1], we examined whether the number of scholarly publications and number of news articles (X) at different time lags were associated with Google search volume (Y). This was performed using a sample cross correlation function (CCF) between a pair of time series at lag *k*, defined as:

$$r_{k}(X,Y)=\frac{\sum \left(X_{t}-\bar{X})(Y_{t-k}-\bar{Y}\right)}{\sqrt{\sum {\left(X_{t}-\bar{X}\right)}^{2}}\sqrt{\sum {\left(Y_{t}-\bar{Y}\right)}^{2}}}$$

in which *X*_*t*_, *Y*_*t*_ are values at time t of each series, and $\bar{X}$, $\bar{Y}$ are the mean values of each series [32]. In our case, the quantity of news or scholarly articles played the role of the X series and Google search volume played the role of the Y series. The CCF would detect time lags at which significant correlation is likely.

A prerequisite to conducting CCF is that the time series is stationary [32, 33]. This means that the mean, variance and autocorrelation structure are independent of time. Results from the Mann-Kendall test (see above) showed evidence trends with time in our data, rendering the time series non-stationary. To correct for this, we conducted first order differencing on both series, in which the time series data is transformed into “differenced data” by taking the changes between two consecutive points in time.

After the two time series were differenced, we performed a prewhitening procedure [33] to separate the linear relationship between the times series from their own autocorrelation to eliminate spurious correlations between time series (see e.g. Probst et al. [34]). Prewhitening was conducted using the function *prewhiten* from R package TSA [27] to yield CCF values for different lags. The CCF value at lag *k* was considered statistically significant if larger than 1.96/√*n* where n is the sample size [32] and such significant lags entered the model in the next step. Since the effect of news reporting on search volume is almost immediate [35], we limited the lags of news articles to within three months.

#### Time series regression

In all regression models conducted in this section, the dependent variable was the benchmark-corrected Google search volume (except for *climate change* in which the benchmark-corrected search volume was log transformed to prevent convergence errors) and the explanatory variables were the significant lags of the number of English news articles and of the number of research articles.

As inspection of autocorrelation function values of the residuals of these models showed serial correlation, we conducted time series regression instead of standard regression to correct for potentially inflated statistical significance [36]. We specified seasonal autoregressive integrated moving average (SARIMA) models for the error processes. The ARIMA(p,d,q) model consisted of the autoregressive (AR) model in which the current value of the series can be explained as a function of *p* past values, the integrated model of order *d*, and the moving average (MA) model in which the current value of the series was explained as a function of current and the *q* most recent past white noise. We developed a SARIMA(p,d,q)(P,D,Q)_s_ model from the ARIMA model by accounting for seasonal fluctuations that occur every *s* seasons, which in our study was 12 months. We started model selection with the basal error model ARIMA(2,0,0)(1,0,0)_12_ for seasonal data [37]. The order of differencing, AR and MA terms were then identified [38].

## Results

### Trend analysis

**Fig 1:**
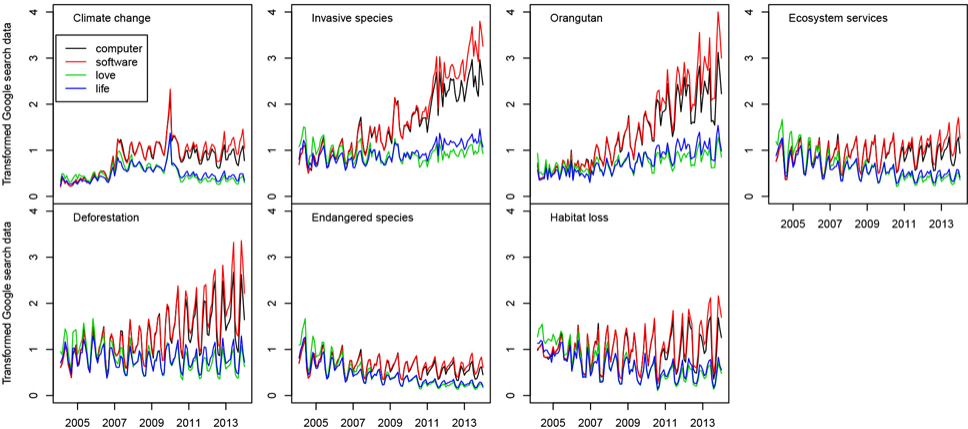
Transformed Google search data for the seven topics studied in 2004–2013 compared to four benchmarks.

Using different benchmark keywords resulted in varying trends for each topic. When *love* was the benchmark, we found significant trends for all topics except for *orangutan* and *deforestation*, of which only *ecosystem services* exhibited an upward trend (Table 1, Fig 1). When *life* was the benchmark, we also found significant trends for five topics: *orangutan*, *ecosystem services*, and *deforestation* exhibited an upward trend while *endangered species* and *habitat loss* exhibited downward trends. When the benchmark was *software*, significant trends were found for all topics, of which only *endangered species* exhibited a downwards trend. When *computer* was the benchmark, we found significant trends for six topics (except for *habitat loss*), of which only *endangered species* exhibited a downward trend. *Ecosystem services* and *endangered species* consistently exhibited a robust upward and downward trend respectively across all four benchmarks. The benchmarks c*omputer*, *software*, *and life* exhibited downward trends when considered singly (tau = -0.998; -0.997; and -0.405 respectively), while *love* exhibited an upward trend (tau = 0.754) (all p-values<0.001). Consequently, stronger upwards trends in the benchmarks led to increased downward trends in conservation keywords, indicating the analyses presented for most of the keywords are sensitive to the choice of benchmark.

**Table 1:** Results of seasonal Mann-Kendall trend test on the search volume in Google for different topics using different benchmark keywords.

Topic	*Love*	*Life*	*Software*	*Computer*
**Climate change**	-0.285 (<0.001)	0.134 (0.0628)	0.604 (<0.0001)	0.313 (<0.0001)
**Orangutan**	-0.107 (0.1342)	0.567 (<0.0001)	0.944 (<0.0001)	0.907 (<0.0001)
**Ecosystem services**	0.618 (<0.0001)	0.827 (<0.0001)	0.916 (<0.0001)	0.888 (<0.0001)
**Deforestation**	-0.107 (0.1343)	0.567 (<0.0001)	0.944 (<0.0001)	0.907 (<0.0001)
**Invasive species**	-0.652 (<0.0001)	-0.022 (0.7567)	0.904 (<0.0001)	0.856 (<0.0001)
**Endangered species**	-0.963 (<0.0001)	-0.944 (<0.0001)	-0.337 (<0.0001)	-0.789 (<0.0001)
**Habitat loss**	-0.736 (<0.0001)	-0.536 (<0.0001)	0.315 (<0.0001)	0.125 (0.0845)

Reported are Kendall’s tau statistic and 2-sided p-value (in brackets).

### Regression model

**Fig 2:**
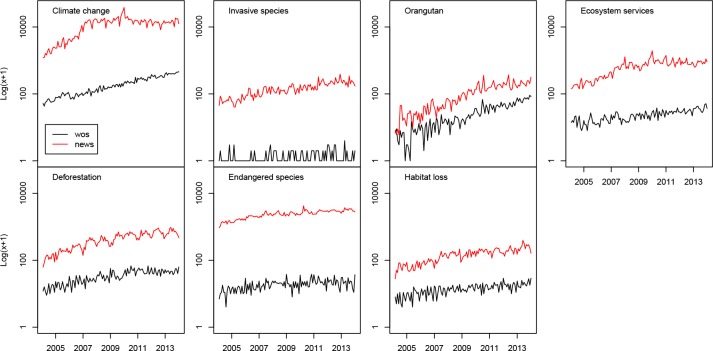
Number of news articles (news) and academic articles recorded in Web of Science (wos) on the seven topics in 2004–2013. Points show total number of articles each months, with values log-transformed.

*Climate change* was the most popular keyword reported in the news with over 1.4 million articles, and *ecosystem services* was the least popular, featuring in 11,870 articles over 10 years. The number of news articles on all topics increased steadily over the 10 years studied (Fig 2) with the fastest increase in number of news articles on *ecosystem services* and the slowest increase for *endangered species*. *Climate change* was also the most popular topic in academic publishing, with over 23,000 articles, and *orangutan* was the least popular with only 47 articles published in the academic fields considered in this study over the 10 years. The number of academic articles increased for all keywords, with *ecosystem services* experiencing the most rapid increase.

Lags of explanatory variables that showed high potential in predicting search volume in Google were identified from the sample cross correlation function (CCF) (Table 2 and S1–S3 Tables in the Supporting Information) and entered the candidate regression models. Across different benchmark keywords, we detected similar lags with the potential to be highly predictive, so these were selected for the regression models: contemporaneous quantity (lag = 0) of news articles for *climate change* and *invasive species;* quantity of news articles three months before Google Trends search volume (lag = -3, where a negative lag indicates months before the observed search volume and a positive lag months after the observed search volume) for *orangutan;* and quantity of scholarly articles 13 months after observed Google Trends search volume (lag = 13) for *deforestation*.

**Table 2:** Results of time series models for each keyword when using *Computer* as the benchmark keyword. Reported are the lags that entered the models; in brackets are their coefficient estimate and standard errors (x10^-3^). Significant lags are in bold.

Keyword	News	Scholarly articles
**Climate change**	**lag = 0 (3.93; 0.43); lag = 1 (0.10; 0.04)**	**lag = -6 (-0.79; 0.28)**
**Orangutan**	**lag = -3 (-2.24; 0.46); lag = 0 (1.64; 0.44)**	**lag = 17 (-68.03; 19.87)**
**Ecosystem services**	lag = -3 (0.69; 0.56); lag = 3 (0.53; 0.51)	–
**Deforestation**	–	lag = 4 (-0.17; 1.02); **lag = 13 (3.00; 0.99)**
**Invasive species**	**lag = 0 (0.81; 0.17)**	–
**Endangered species**	–	lag = -7 (0.21; 0.90); lag = 4 (-0.89; 0.83); lag = 5 (0.20; 0.94); lag = 16 (-0.83; 0.86); lag = 17(-0.68; 0.87)
**Habitat loss**	lag = 1 (5.58; 4.48)	lag = 2 (6.47; 4.47)

The results for the other three benchmark keywords are available in S1–S3 Tables in the supporting information. The news variables for climate change have been back-transformed. Significant lags are in bold. Lag = 0 means contemporaneous effect of scholar articles; negative or positive lags mean the quantity of news/scholarly articles published before the observed Google search volumes, respectively.

The SARIMA models showed a pattern of positive and contemporaneous correlation between the number of news articles and search volumes for *climate change* (all benchmarks), *orangutan* (all benchmarks), and *invasive species* (all benchmarks except *life*). Other than this general trend, four main groups of results could be observed.

First, the benchmark-corrected search volume and the number of news or scholarly articles were positively correlated with a negative time lag (negative lags mean the changes in search volume may take place later than the changes in the number of news or scholarly articles). This result occurs when the changes in the number of news or scholarly articles may drive the changes in the search volume with a time delay. However, this only occurred for *orangutan* with lag = -3 of news articles (with *software* and *computer* as benchmarks, Table 2 and S2 Table) and *deforestation* with lag = -3 of scholarly articles (with *life* as benchmark, S1 Table).

Second, we also observed negative time lags and negative correlation between the benchmark-corrected search volume and the number of news or scholarly articles. This result occurs when there is an increase in the number of news or scholarly articles followed by a decrease in the search volume, or vice-versa with a time delay. We observed this in our research for *orangutan* with lag = -3 for news articles (*software* and *computer* as benchmarks, Table 2 and S2 Table), *climate change* with lag = -6 for scholarly articles (*computer* as benchmark, Table 2), and *deforestation* with lag = -3 also for scholarly articles (*life* as benchmark, S1 Table).

Third, the benchmark-corrected search volume and the number of news or scholarly articles were positively correlated with a positive time lag. This occurs when the number of news or scholarly articles increase and search volume increases, or vice-versa, in a previous point in time (meaning the changes in the number news or scholarly articles take place later than the changes in search volume). This significant correlation between benchmark-corrected search volume and news articles for *climate change* at positive lag = 1 (benchmarks *computer* and *love*, Table 2 and S3 Table), for *ecosystem services* at positive lag = 3 (benchmark: *life*, S1 Table), suggests that news reporting may be responsive to events that are already generating online interest in these fields rather than leading public interest, e.g. a climate change convention may generate news coverage that extends in time while public interest wanes. However, for both keywords, such delayed correlation presents a lower effect size than the contemporaneous effect or negative lag (S1–S3 Tables). Similarly, the significant correlation between benchmark-corrected search volume and scholarly articles for *deforestation* at positive lag = 13 (all benchmarks except for *love*, Table 2) and lag = 4 (benchmark: *software*, S2 Table), suggests that scholarly work may be responsive to, rather than leading public interest.

Fourth, we also observed that the benchmark-corrected search volume and the number of news or scholarly articles were negatively correlated with a positive time lag. This result occurs when the number of news or scholarly articles decreases when search volume increases, or vice-versa, in a previous point in time. This was observed for *climate change* with lag = 5 for scholarly articles (*life* as benchmark) and *orangutan* with lag = 17 also for scholarly articles (*software* and *computer* as benchmarks). However, this type of correlation is difficult to interpret on theoretical grounds and more research is needed to validate its persistence.

## Discussion

Our results have shown that the trends of interest in conservation topics are sensitive to the benchmark chosen, therefore raw data from Google Trends should not be interpreted literally. To confidently identify the trend in searches for a certain topic, its search volume should be compared to benchmarks. In contrast for previous studies using Google search volume as a proxy of public interest [12, 26], we did not find that, in general, public interest towards environmental topics is declining. Of the seven conservation topics considered in this study, we found an upward trend in online public interest in *ecosystem services* and a downward trend in four other topics when using *love* (the most conservative benchmark). With less conservative benchmarks we found: upward trends for three keywords and downward trends for two keywords (*life*); a downward trend for only *endangered species*, and upward trends for six and five topics respectively when using the purportedly neutral keywords of *software* and *computer* as benchmarks. In a previous study on 19 broad environmental topics, Mccallum and Bury [12] found a declining trend for 16 topics and an increasing trend for three topics, which suggested a general waning of public interest in environmental topics. Of the three keywords (*ecosystem services*, *endangered species*, *invasive species*) that overlap in our analysis with Mccallum and Bury [12], we obtained similar results only for *endangered species*. On the other hand, our upward trend results also agree with those in Proulx et al. [39] for *ecosystem services*.

The implications of our findings are not to substantiate that public interest is following a specific trend, but to suggest scholars to exercise caution when generalizing public interest trends in conservation topics, in particular when using Google Trends data. The discrepancy between our findings and the declining trends found by Mccallum and Bury [12] could be due to the different set of topics explored, but where the keywords overlap the differences can be attributed to our use of benchmark keywords to correct for shifting baselines in search volume. Another caveats of using Google Trends data is that over a broad temporal scale, changes may not be unidirectional but instead demonstrate peaks and troughs [4]. This may be due to seminal events [35], for example, public interest in conservation has been found to peak following an extinction event, although this effect is transient [10]. It is also possible that interest within conservation follows the issue-attention cycle model, where a novel issue is initially enthusiastically attended to by the public, with this fleeting stage followed by a gradual decline in public interest and a shift of interest to new topics [40]. Individual topics could follow different trajectories as more novel topics such as *ecosystem services* attract more attention from the media and the public.

We incorporated benchmark keywords to address the natural shifting baseline whereby Google search terms decrease [13]. Since absolute search volume in Google is undisclosed, for any topic the validity of analyses on trends of public interest using Google Trends rests on the assumption of stable search volume for selected benchmark keywords. To be conservative in our analysis, we chose 4 different benchmarks and found a high sensitivity of benchmark choice for the trends identified.

The main limitation of our analyses is that it is restricted to internet users that are conducting Goole searches in English. Even though English remains to be the most popular official language globally, different languages and cultures could have different conservation concerns [26]. In addition, other search engines could also be more popular than Google Trends in certain countries, e.g. Baidu is the main search engine in China [41]. Local-level conservation issues could also affect the relative search volume: data for regional interest in Google Trends suggested that *invasive species* attracts public attention mostly in North America and Australia where the impacts of biological invasion has been well-documented while the online public in tropical countries could be more interested in the topic of *deforestation* compared to those in non-tropical regions.

To fully understand the dynamics of public interest on environmental topics, we suggest a broader set of topics should be explored using a variety of different benchmarks. At the very least, conservation scientists can compare interest levels in different conservation topics for Google users, or using benchmarks. Understanding the relative popularity of different conservation topics, rather than attempting to assess absolute trends, could assist the prioritization of communication efforts. For example, trends analysis in our study suggested that public attention in *endangered species* is declining relative to interest in *ecosystem services*. Web-based public outreach programs on *endangered species* could therefore use this knowledge to integrate or foster the *ecosystem services* component in their agenda. In addition, the upward trend observed for *ecosystem services* in this study is likely to be robust to most neutral benchmark keywords. An upward trend in search volume is further supported by the fact that the field of *ecosystem services* has experienced the most rapid increase in news reporting, academic publishing and has been promoted in international policy [42]. Future analyses could use the keyword “ecosystem services” as an upper bound against which the changes in the popularity of other conservation topics are evaluated.

The link between search volume and news reporting was positive for *climate change*, *orangutan*, *ecosystem services* and *invasive species* for most benchmark terms considered. It is however difficult to establish the direction of causality. The synchrony of news reporting and search volume could also show a reflective fluctuation in the level of public interest in environmental topics. Such a reflective relationship has been described in the literature. For example Phillis et al. [1] used news articles as a proxy for public interest. On the other hand, this relationship could also be produced if there is an immediate impact of news reporting on public interest, or even on framing public perceptions, as has been proposed in agenda-setting theory [43]. Overall, our findings support the link between media and the public in conservation [44] and therefore the need to include news media as a separate sector in the conservation process beside the public, scientists and policy makers [5].

The relationship between academic publishing and online public interest was in general represented by positive lags. This could be due to researchers responding to events of public interest, such as natural disasters or major conservation policies that imply a necessary lag to perform the research and go through the review and publication process. The rest of the correlation terms showed inconclusive patterns within the time period studied. Even though we found a positive correlation for *deforestation*, the overall relationship appears to be negative for the keywords *climate change* and *orangutan*. No significant relationship was detected for the remaining topics. Even though previous studies have suggested a correlation between public interest and academic work [22, 24], such conclusion is often presumed rather than quantitatively proven. In this study we could not detect a strong and consistent correlation between Google Trends search volume and academic publishing. This could be due to the content and delivery of specific articles, rather than absolute volume of research articles, is the key determinant in attracting public attention. For example, a single research article on an emotive issue or significant event such as species extinction [10] may spark greater interest than numerous non-sensational stories. Relatedly, only a small fraction of research articles generate interest and only a few articles are reported by multiple news outlets with diverse audiences [5]. Since many scholarly journals are not available to the general public, audiences for academic articles may be smaller and less diverse [45] and thus less difficult to detect using overall search volumes in Google Trends. In addition, it is also possible that open-access articles or articles followed by press releases written for the general public could elicit more online searches. This could explain the negative correlations between number of scholarly articles and search volume observed, as the public may interact differently with journals with different accessibility. Future research considering the characteristics of each journal should be directed to further understand how research articles interactive with online search volume.

## Conclusion

Google Trends data are a powerful tool to monitor and evaluate public interest in conservation. By conducting keyword comparisons within the discipline of conservation, we may gain insight on changing public interests and use this knowledge to prioritize communication about conservation to the public. To overcome the issue of shifting baselines in Google Trends data, we suggest conservation scientists explore the relative popularity of different conservation topics by comparing them to each other, or to other topics with increasing interest, such as *ecosystem services*. Our discovery of the stronger relationship between news articles and public interest, in comparison with the relationship between public interest and research articles, highlights the immediate influence of mass media conservation issues in the public sphere, and the lag between time taken to conduct research and publish.

## Supporting Information

S1 File Dataset of the analysis. (CSV)

S1 Table Results of SARIMA models for each keyword when using *life* as the benchmark keyword. Reported are the lags that entered the models; in brackets are their coefficient estimate and standard errors (x10^-3^). The news variables for climate change have been back-transformed. Significant lags are in bold.
(DOCX)

S2 Table Results of SARIMA models for each keyword when using *software* as the benchmark keyword. Reported are the lags that entered the models; in brackets are their coefficient estimate and standard errors (x10^-3^). The news variables for climate change have been back-transformed. Significant lags are in bold.
(DOCX)

S3 Table Results of SARIMA models for each keyword when using *love* as the benchmark keyword. Reported are the lags that entered the models; in brackets are their coefficient estimate and standard errors (x10^-3^). The news variables for climate change have been back-transformed. Significant lags are in bold.
(DOCX)
