# Supplementary material for: Analysis of the Capacity of Google Trends to Measure Interest in Conservation Topics and the Role of Online News
Source: PLoS One. 2016 Mar 30;11(3):e0152802. doi: 10.1371/journal.pone.0152802 (PMC4814066; doi:10.1371/journal.pone.0152802)
Supplement: S1 Table — Reported are the lags that entered the models; in brackets are their coefficient estimate and standard errors (x10-3). The news variables for climate change have been back-transformed. Significant lags are in bold. (DOCX) [file pone.0152802.s002.docx]

S1. Results of SARIMA models for each keyword when using *life* as the benchmark keyword. Reported are the lags that entered the models; in brackets are their coefficient estimate and standard errors (x10^-3^). The news variables for climate change have been back-transformed. Significant lags are in bold. Lag= 0 means contemporaneous effect of scholar articles; negative or positive lags means the quantity of news/scholarly articles published before or after the observed Google search volumes.

| Keyword | News | Scholarly articles |
| --- | --- | --- |
| Climate change | **lag= 0 (1.84; 0.26)**  lag= 1 (0.44; 0.27) | **lag= 5 (-0.81; 0.19)** |
| Orangutan | lag= -3 (-0.09; 0.27)  **lag= 0 (0.79; 0.26)** | – |
| Ecosystem services | **lag= -3 (1.12; 0.30)**  **lag= 3 (1.07; 0.28)** | – |
| Deforestation | – | lag= -**3 (-3.32; 0.77)**  **lag= 13 (1.53; 0.72)** |
| Invasive species | lag= 0 (0.18; 0.12)  lag= 2 (-0.14; 0.10) | lag= 13 (-1.51; 0.95) |
| Endangered species | – | lag= -7 (-0.79;0.74)  lag= 4 (-0.78; 0.75)  lag= 5 (0.21; 0.86)  lag= 16 (-0.06; 0.76)  lag= 17 (0.20; 0.78) |
| Habitat loss | lag= 1 (-3.13; 3.58) | – |
