# Supplementary material for: Analysis of the Capacity of Google Trends to Measure Interest in Conservation Topics and the Role of Online News
Source: PLoS One. 2016 Mar 30;11(3):e0152802. doi: 10.1371/journal.pone.0152802 (PMC4814066; doi:10.1371/journal.pone.0152802)
Supplement: S2 Table — Reported are the lags that entered the models; in brackets are their coefficient estimate and standard errors (x10-3). The news variables for climate change have been back-transformed. Significant lags are in bold. (DOCX) [file pone.0152802.s003.docx]

S2. Results of SARIMA models for each keyword when using *software* as the benchmark keyword. Reported are the lags that entered the models; in brackets are their coefficient estimate and standard errors (x10^-3^). The news variables for climate change have been back-transformed. Significant lags are in bold. Lag= 0 means contemporaneous effect of scholar articles; negative or positive lags means the quantity of news/scholarly articles published before or after the observed Google search volumes.

| Keyword | News | Scholarly articles |
| --- | --- | --- |
| Climate change | **lag= 0 (3.73; 0.44)**  lag= 1 (0.59; 0.44) | – |
| Orangutan | **lag= -3 (-2.46; 0.51)**  **lag= 0 (1.72; 0.50)** | **lag= 17 (-62.35; 22.26)** |
| Ecosystem services | lag= -3 (0.53; 0.72)  lag= 3 (0.56; 0.60) | lag= -7 (4.84; 2.83) |
| Deforestation | – | **lag= 4 (2.40; 1.10)**  **lag= 13 (5.04; 1.32)** |
| Invasive species | **lag= 0 (0.69; 0.19)**  lag= 2 (0.19; 0.19) | – |
| Endangered species | – | lag= -7 (-0.15; 0.91)  lag= 4 (-0.86; 0.84)  lag= 5 (0.29; 0.96)  lag= 16 (-0.96; 0.89)  lag= 17 (-0.60; 0.90) |
| Habitat loss | lag= 1 (5.97; 5.18) | lag= 2 (9.70; 5.33)  lag= 14 (4.76; 5.29) |
