# Supplementary material for: Analysis of the Capacity of Google Trends to Measure Interest in Conservation Topics and the Role of Online News
Source: PLoS One. 2016 Mar 30;11(3):e0152802. doi: 10.1371/journal.pone.0152802 (PMC4814066; doi:10.1371/journal.pone.0152802)
Supplement: S3 Table — Reported are the lags that entered the models; in brackets are their coefficient estimate and standard errors (x10-3). The news variables for climate change have been back-transformed. Significant lags are in bold. (DOCX) [file pone.0152802.s004.docx]

S3. Results of SARIMA models for each keyword when using *love* as the benchmark keyword. Reported are the lags that entered the models; in brackets are their coefficient estimate and standard errors (x10^-3^). The news variables for climate change have been back-transformed. Significant lags are in bold. Lag= 0 means contemporaneous effect of scholar articles; negative or positive lags means the quantity of news/scholarly articles published before or after the observed Google search volumes.

| Keyword | News | Scholarly articles |
| --- | --- | --- |
| Climate change | **lag= 0 (2.69; 0.34)**  **lag= 1 (1.08; 0.33)** | — |
| Orangutan | lag= -3 (-0.38; 0.26)  **lag= 0 (0.59; 0.26)**  lag= 1 (-0.10; 0.26) | — |
| Ecosystem services | **lag= -3** **(0.66; 0.28)**  lag=3 (0.52; 0.26) | — |
| Deforestation | — | lag=1 (-0.08; 1.02)  lag=13 (-0.23; 1.04) |
| Invasive species | **lag= 0 (0.19; 0.02)** | — |
| Endangered species | — | lag=5 (0.27; 0.82)  lag=17 (0.53; 0.75) |
| Habitat loss | lag= 1 (-0.26; 3.67) | — |
